# Structural Interaction and Functional Regulation of Polycystin-2 by Filamin

**DOI:** 10.1371/journal.pone.0040448

**Published:** 2012-07-10

**Authors:** Qian Wang, Xiao-Qing Dai, Qiang Li, Zuocheng Wang, María del Rocío Cantero, Shu Li, Ji Shen, Jian-Cheng Tu, Horacio Cantiello, Xing-Zhen Chen

**Affiliations:** 1 Membrane Protein Disease Research Group, Department of Physiology, University of Alberta, Edmonton, Alberta, Canada; 2 Cátedra de Biofísica, Facultad de Odontología, Universidad de Buenos Aires, Buenos Aires, Argentina; 3 Zhongnan Hospital, Wuhan University, Wuhan, Hubei, China; 4 Nephrology Division, Department of Medicine, Massachusetts General Hospital and Harvard Medical School, Charlestown, Massachusetts, United States of America; Emory University, United States of America

## Abstract

Filamins are important actin cross-linking proteins implicated in scaffolding, membrane stabilization and signal transduction, through interaction with ion channels, receptors and signaling proteins. Here we report the physical and functional interaction between filamins and polycystin-2, a TRP-type cation channel mutated in 10–15% patients with autosomal dominant polycystic kidney disease. Yeast two-hybrid and GST pull-down experiments demonstrated that the C-termini of filamin isoforms A, B and C directly bind to both the intracellular N- and C-termini of polycystin-2. Reciprocal co-immunoprecipitation experiments showed that endogenous polycystin-2 and filamins are in the same complexes in renal epithelial cells and human melanoma A7 cells. We then examined the effect of filamin on polycystin-2 channel function by electrophysiology studies with a lipid bilayer reconstitution system and found that filamin-A substantially inhibits polycystin-2 channel activity. Our study indicates that filamins are important regulators of polycystin-2 channel function, and further links actin cytoskeletal dynamics to the regulation of this channel protein.

## Introduction

**Figure 1:**
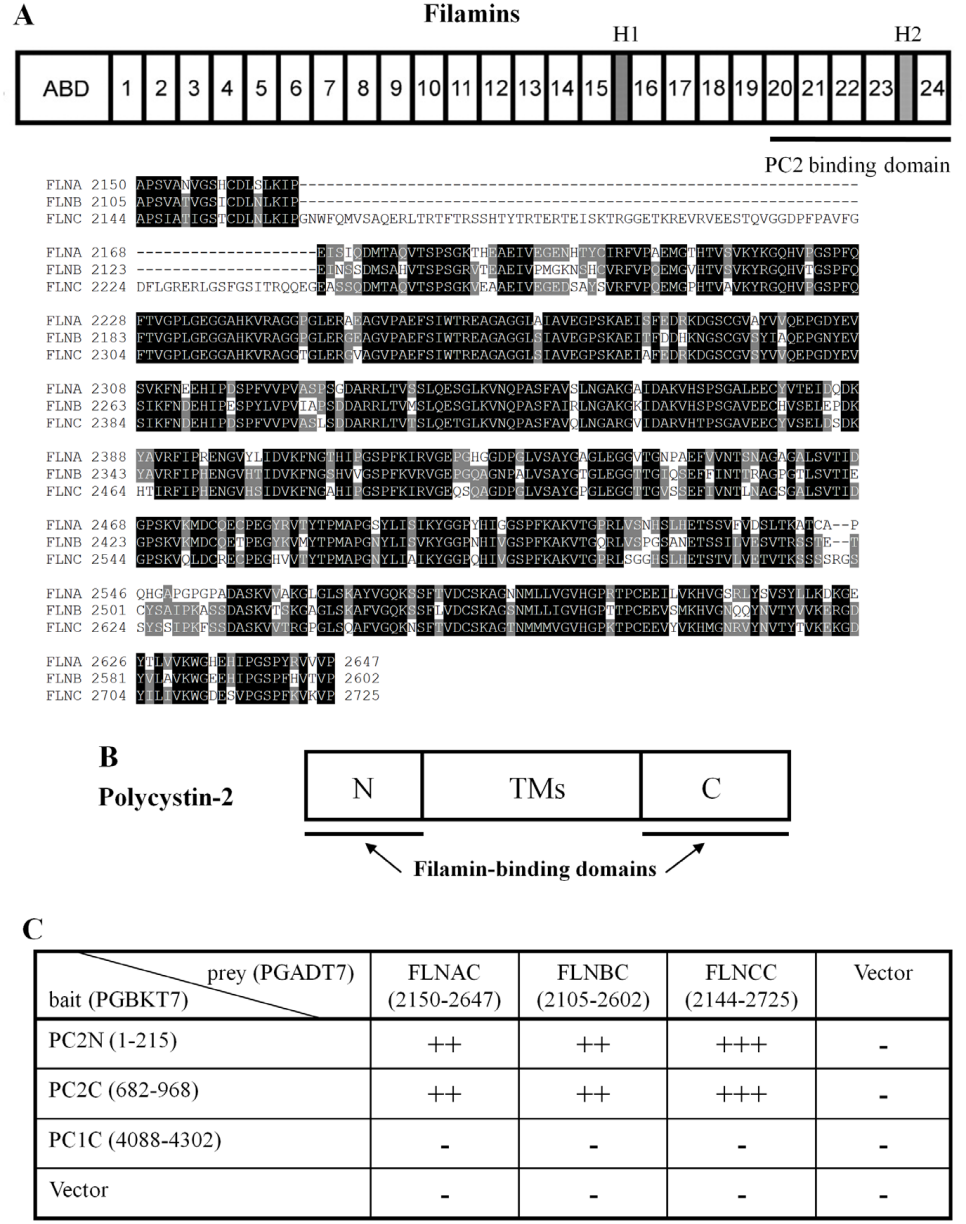
Structural domains and sequence of FLNs and PC2, and their interaction identified by yeast two-hybrid assay. (**A**) Upper panel, domain structure of human filamins (FLNs), FLNA, FLNB and FLNC, and sequence alignment. FLNs share common features such as the N-terminal actin-binding domain (ABD) and a semi-rigid rod composed of 24 Ig-like repeats (∼96 aa each) interrupted by two short flexible hinges (H1 and H2). The PC2 binding domain is indicated. Lower panel, sequence alignment for FLNs C-terminus, which share 70–75% sequence similarity. (**B**) Structural domain of PC2 is shown, indicating both the intracellular N- and C- termini (PC2N and PC2C, FLN-interacting domains) and transmembrane spans (TMs). (**C**) Interaction data revealed by β-GAL induction assay in the yeast two-hybrid screen system. PC2N, PC2C, the C-terminus of polycystin-1 (PC1C) and the empty vector were used as bait. The C-termini of FLNs and the empty vector were used as prey. A bait-prey pair was co-transformed in the yeast strain Y187, and the β-GAL activity was determined based on the time when colonies turned blue in X-gal filter lift assays. “+++”, “++”, “+” and “–” indicate development of blue color within 1, 3 and 24 hours, and no development of blue color within 24 hours, respectively.

Mammalian filamin (FLN) was first isolated from rabbit macrophages in 1975 as an actin-binding protein [1]. The mammalian FLN family consists of three ∼280-kDa isoforms, filamin-A (FLNA), -B (FLNB) and -C (FLNC), sharing 60–80% whole sequence homology, of which FLNA is the most abundant and widely distributed [2]. FLNs contain an N-terminal actin-binding domain (ABD) that shares sequence similarity with other actin-binding proteins, and a rod domain consisting of 24 repeated anti-parallel β-sheets with one or two short ‘hinges’ inserted before repeats 16 and 24 (Fig. 1A). FLNs self-associate within a C-terminal 7-kDa domain, to form homodimers with flexible V-shaped structures acting as ‘a molecular leaf spring’ to facilitate cross-linking of actin filaments [3].

By cross-linking actin filaments at wide angles, FLNs act as important actin cytoskeleton organizers implicated in sol-gel transformations and membrane stabilization as anchors of many transmembrane proteins, and as scaffolding proteins for various signaling molecules [4]. Indeed, as versatile scaffolding proteins, FLNs physically interact with, and help regulating the activity of, many proteins with diverse functions [2]. Mutations in the FLNA and FLNB genes are known to cause a variety of developmental disorders in humans, including bone anomalies, periventricular heterotopia, aortic dissection and aneurysm [5]–[7].

Polycystin-2 (PC2), also known as TRPP2, is a member of the transient receptor potential polycystin (TRPP) subfamily of TRP channels. PC2, encoded by the *PKD2* gene, is a 968 amino-acid (aa) integral membrane protein with six transmembrane domains and intracellularly localized N- and C-termini. PC2 bears similar membrane topology with other TRP channels and voltage-gated cation channels [8]. PC2 localizes to different subcellular compartments such as the endoplasmic reticulum (ER) membrane [9], the primary cilium [10], [11] and the plasma membrane (PM) [12]. Mutations in *PKD2* account for 10–15% of patients with autosomal dominant polycystic kidney disease (ADPKD), a common genetic disorder with a population prevalence of ∼1∶1000 that is characterized by formation of cysts in various organs, including kidneys, liver and pancreas [13]. Non-cystic manifestations of the disease include mitral valve prolapse, aortic dissection and vascular aneurysm [14], [15]. Despite the fact that cystic cells are associated with cell over-proliferation, de-differentiation and apoptosis, the underlying mechanisms of cyst formation remain ill-defined. We found that PC2 inhibits cell proliferation by up-regulating the activity of the translation inhibitor eIF2α [16]. Mice with either loss- or gain-of-function of PC2 are both cystogenic [17], [18]. Thus, it seems critical for cells to control the PC2 cellular expression level within a narrow range. We recently found that PC2 degradation is regulated by the ER-associated degradation (ERAD) pathway through the ubiquitin-proteasome system demonstrating that PC2 is a novel ERAD substrate [19].

There are important connections between PC2 and the actin cytoskeleton. About half of PC2 interacting partners identified to date are cytoskeleton or cytoskeleton-associated proteins [20]. PC2 interacts with α-actinin, an actin-bundling protein important in cytoskeletal organization, cell adhesion, proliferation and migration. Interestingly, both intracellular N- and C-termini of PC2 associate with this actin-binding protein. This interaction substantially increases PC2 channel activity by increasing the channel’s open probability, but not its single-channel conductance, as shown using preparations containing either the isolated PC2 protein or PC2 from human placenta [21]. Thus, α-actinin binds to the PC2 channel to regulate its gating rather than affecting its physical channel pore. Dynamic changes in actin filament organization also modulate PC2 channel function in the apical membrane of the human syncytiotrophoblast, a preparation that contains abundant endogenous PC2 [22]. Either addition of G-actin, treatment with the actin-filament disrupter cytochalasin D, or addition of the actin-severing protein gelsolin to the apical membrane of the human syncytiotrophoblast (hST) dramatically increases PC2 channel activity [23]. Thus, the actin cytoskeleton anchors PC2 to the PM not only for structural purposes, but also to regulate its channel function in a way that could be as complex as its own structure. Further evidence supporting the actin cytoskeleton as an integral part of a sensor implicating PC2 was provided by using apical membrane vesicles from hST. It was reported that both hydrostatic and osmotic pressures stimulate PC2 channel activity in hST, a phenomenon in which the effect of both physical factors was abolished by pre-treatment with the cytoskeletal disrupter cytochalasin D [24]. Thus, PC2 and actin structures together, but not the channel alone, confer PC2 sensitivity to these physical factors. This suggests that the actin cytoskeleton associated with PC2 acts as an integral part of a sensing complex responsive to hydrostatic and osmotic changes.

In the present study, we first examined and documented by various *in vitro* and *in vivo* approaches, the physical interaction between PC2 and the actin cross-linking proteins FLNs. We then examined the effect of FLNs on PC2 channel function.

## Materials and Methods

### Antibodies

Three anti-PC2 antibodies were used in this study, including mouse 1A11 [21], [25], goat G-20 [21] and rabbit H-280 (Santa Cruz Biotech, Santa Cruz, CA). The antibodies used to label FLNs included mouse FIL-2, raised using chicken gizzard FLN antigen (Sigma-Aldrich Canada, Oakville, ON), mouse anti-FLNA E-3 and rabbit H-300 (Santa Cruz Biotech). Affinity purified goat anti-GFP EU4 (Eusera, Edmonton, AB) was utilized for immunoprecipitation (IP) and mouse anti-GFP B-2 (Santa Cruz Biotech) for immunoblotting (IB). Mouse anti-His Tag 27E8 (New England Biolabs, Pickering, ON) was employed to detect His-tagged FLNs C-termini in GST pull-down. Rabbit anti-calnexin C4731 (Sigma-Aldrich Canada) was used for immunofluorescence (IF). Either rabbit A2066 (Sigma-Aldrich Canada) or mouse anti-β-actin C4 (Santa Cruz Biotech), and mouse anti-HSP60 H-1 (Santa Cruz Biotech) antibodies were used as loading controls. Secondary antibodies were purchased from GE Healthcare (Baie d’Urfe, Quebec) or Santa Cruz Biotech.

### Plasmid Construction

The FLNAC and FLNBC cDNAs were isolated by PCR from either a human kidney cDNA library or human embryonic kidney (HEK) 293T cells. The cDNA encoding the C-terminus of FLNC (FLNCC, A2144-P2725) was cut from the pACT2-FLNCC plasmid. The cDNAs were subcloned into pGADT7 and pET28a (Novagen, EMD Chemicals, Gibbstown, NJ) for yeast and bacterial expression, respectively. Mammalian expression plasmids pEGFP-PC2, pEGFP-PC2 ΔC (aa 1-688, lacking the C-terminus) and pEGFP-PC2ΔN (aa 209-968, lacking the N-terminus), in which GFP is fused to the N-terminal end of PC2, were constructed based on a method previously described [21]. All plasmid construction and cDNA sequences were verified by sequencing.

### Yeast Two-hybrid Analysis

cDNA fragments encoding either the N-terminus (PC2N; amino acid 1-215) or C-terminus (PC2C; amino acid 682-968) of human PC2 were subcloned in frame into the GAL4 DNA binding domain of the pGBKT7 vector (Clontech, Palo Alto, CA) by a PCR-based approach. Either PC2N or PC2C was used as a bait in a yeast two-hybrid screen using human heart library (Clontech) constructed in the pGADT7 vector in the yeast strain AH109 containing *Ade2*, *His3* and *LacZ* reporter genes under the control of the GAL4 upstream activating sequences as described previously [21]. A pair bait-prey was then co-transformed in the yeast strain Y187. The β-GAL activity was determined based on the time it took for colonies to turn blue in X-gal filter lift assays performed at 30°C.

### GST Pull-down

The cDNA fragments encoding PC2N or PC2C were subcloned into the pGEX5X vector (Pharmacia, Piscataway, NJ, USA). Expression of either GST-PC2C, GST-PC2N or GST alone was conducted in the protease-deficient bacterial strain *E. coli* BL21 (DE3). Protein expression was allowed for 5 hours at 28°C after inducing with IPTG (1 mM). The bacterial pellet was obtained and then lysed by grinding with Alumina type A-5 (Sigma-Aldrich, Canada) in an extraction buffer, containing: 140 mM NaCl, 10 mM Na_2_HPO_4_, and 1.8 mM KH_2_PO_4_, pH 7.5. The supernatant was either used for GST purification with a commercial kit (Clontech, Polo Alto, CA), or used directly in GST pull-down experiments. The cDNA fragments encoding either, FLNAC, FLNBC, or FLNCC were cloned into the pET28a vector containing a poly His epitope on its 5′ end (Novagen). Proteins were similarly expressed as GST fusion complexes purified by a His Bind® kit (Novagen) according to manufacturer’s protocol. Either pre-cleared bacterial protein extracts (250 µl) containing GST-tagged PC2N, PC2C or GST alone, or a purified GST fusion protein (2 µg), were incubated with purified His-FLNAC, -FLNBC or -FLNCC fusion protein (2 µg), in binding buffer, containing: 150 mM NaCl, 1.0 mM CaCl_2_, and 50 mM Tris, pH 7.5. The mixture was incubated at room temperature (RT) for 1 hour with gentle shaking, followed by another hour of incubation after addition of 100 µl glutathione-agarose beads (Sigma-Aldrich Canada). The beads were then washed 4–5 times with 140 mM NaCl, 10 mM Na_2_HPO_4_, 1.8 mM KH_2_PO_4_, pH 7.5 and the remaining proteins eluted using 10 mM glutathione, 50 mM Tris, pH 8.0. The protein samples were then prepared for IB.

### Human Melanoma Cell Lines

Human melanoma M2 cells, grown as previously described [26], is deficient of FLNA. M2 cells display impaired motility and a dysfunctional actin organization. Transfection of FLNA into M2 cells generated A7 cells. FLNA-replete A7 cells that exhibit both normal motility and actin cytoskeletal organization [26]. To generate M2 and A7 PC2 stable cell lines, 600 mg/ml of hygromycin and G418 (Invitrogen Canada Inc.) were added to select viable clones one recovery day following transfection, and then maintained using hygromycin (100 µg/ml) or hygromycin plus G418 (300 µg/ml), respectively.

### Cell Culture and Transfection

Renal cell lines, including HEK293T, Madin-Darby canine kidney (MDCK), inner medullary collecting duct (IMCD), and porcine kidney cells LLC-PK1 were cultured in Dulbecco’s modified Eagle’s medium (DMEM) supplemented with L-glutamine, penicillin-streptomycin, and 10% fetal bovine serum (FBS). MDCK cells stably expressing either GFP-PC2 or GFP alone, were selected as previously described [27] and maintained in the presence of G418 (300 µg/ml). The human melanoma cell lines M2 and A7 devoid of, and stably replete with, filamin A, respectively [26], were maintained in minimal essential medium supplemented with 8% newborn calf serum and 2% fetal calf serum. Transfection of cDNAs was performed using Lipofectamine 2000 (Invitrogen Canada Inc., Burlington, ON) according to the manufacturer’s protocol.

### Immunofluorescent Microscopy

MDCK, M2 (FLNA-deficient) and A7 (FLNA-replete) human melanoma cells stably expressing PC2 were grown on coverslips, fixed for 10 minutes at RT with 2% paraformaldehyde, and washed twice with PBS. Cells were then permeabilized for 3 minutes at RT with PBS containing 0.05% Triton X-100, blocked in PBS with 3% skim milk powder for 1 hour, and incubated with either anti-FLNA E3 or anti-calnexin overnight at 4°C, followed by 1 hour incubation with the secondary antibody. Cells were finally washed with PBS containing 0.1% Tween 20. Vectashield mounting medium with DAPI (Vector Laboratories, Burlingame, CA) was used to protect IF signals from fading. Pictures were captured with a fluorescence microscope with Colibri LED (Carl Zeiss Canada Ltd., Toronto, ON). The final composite images were created using AxioVision 4.8 (Carl Zeiss Canada Ltd.).

### Protein Preparation and Lipid Bilayer Electrophysiology

Commercial chicken gizzard filamin (FLNA, Cell Sciences, Canton, MA) was used as a modulator of PC2 channel function. Purified PC2 protein was obtained either by *in vitro* translation (Applied Biosystems) or by our modified tandem affinity purification method from PC2 stably expressing MDCK cells [21], [27]. PC2-containing human syncytiotrophoblast (hST) apical membrane vesicles were prepared and used as previously described [22]. PC2-containing preparations were reconstituted in a lipid bilayer system for electrophysiology studies, as previously described [27], [28]. Briefly, a lipid bilayer membrane was formed with a mixture of 1-palmitoyl-2-oleoyl phosphatidyl-choline and phosphatidyl-ethanolamine (Avanti Polar Lipids, Birmingham, AL, USA) at a 7∶3 ratio in a Deldrin cup inserted in an acrylic chamber (Harvard Apparatus, Montreal, QC, Canada). The PC2 preparation was either added to the *cis* chamber in the proximity of the bilayer, or was “painted” directly into the membrane. FLN was added to the *cis* chamber of the bilayer cuvette, to a final concentration of approximately 25 nM. Negative controls were also conducted by addition of either a similar volume of saline without FLN, or addition of the same concentration of denatured FLN obtained by boiling the protein for 5–10 minutes. Voltage clamping of single channel currents was performed using Clampex 9 (Molecular Devices, Union City, CA, USA).

### Data Analysis

IB signals were quantified by ImageJ (National Institute of Health, Bethesda, MD), analyzed and plotted using SigmaPlot 11 (**Systat Software Inc.,** San Jose, CA). Data were expressed as mean ± SEM (N), where N indicates the number of experimental repeats. Statistical analysis was conducted by Student’s t-test, and a probability value (p) of less than 0.05 was considered significant (*).

**Figure 2 pone-0040448-g002:**
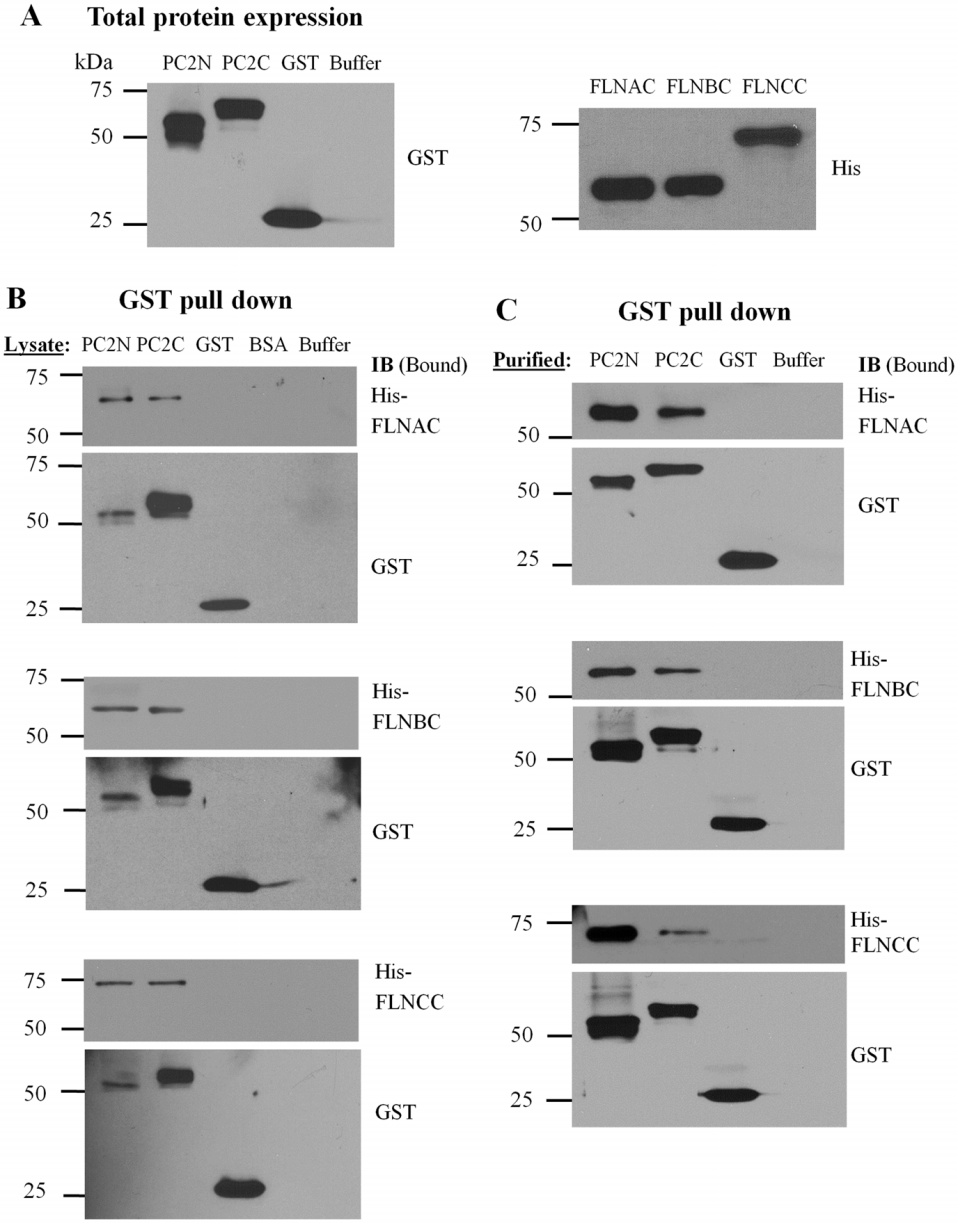
Interaction between PC2 and filamins by GST pull-down. (**A**) Total expression of purified GST- or His-tagged proteins. Left panel, representative data obtained with an anti-GST antibody showing purified GST fusion proteins and buffer from *E. coli*. Right panel, IB imaging obtained with an anti-His antibody showing purified His-tagged FLNAC, FLNBC and FLNCC from *E. coli*. (**B**) *E. coli* lysates from cells expressing GST-PC2N, GST-PC2C, GST alone, BSA or binding buffer alone, were incubated with purified His-tagged FLNAC, FLNBC or FLNCC. Glutathione-agarose beads were used to pull down GST epitope binding proteins. The resultant (Bound) protein samples were immunoblotted with an anti-His antibody (upper panels) or an anti-GST antibody (lower panels) to indicate the effective GST fusion proteins participated in interaction with His-tagged proteins. Data are representative of six experiments. (**C**) Data were obtained under similar conditions as in panel B, except that we utilized purified GST-PC2N, GST-PC2C, GST alone, or the binding buffer alone, incubated with purified His-tagged FLNAC, FLNBC or FLNCC. Data are representative of six experiments.

## Results

### Association of PC2 with Filamins Revealed by Yeast Two-hybrid Assay

To identify novel proteins interacting with PC2 *in vivo*, we screened a human heart yeast two-hybrid library (Clontech) with the PC2 N-terminus (PC2N, aa M1-K215) and C-terminus (PC2C, aa D682-V968), as previously described [21], [29]. One plasmid isolated from the library represented a splicing variant of FLNC (Accession Number: AF146692, 9044 bp coding for 2691 aa). The identified FLNC cDNA was the 3′ fragment starting at nucleotide G6331, encoding a polypeptide that corresponds to FLNC aa A2111-P2691 and comprises both the 20–24 repeats and the second hinge. This region of FLN interacted with both PC2N and PC2C (Fig. 1). Given that the three mammalian FLN isoforms share high sequence similarities, we further explored whether FLNA and FLNB, which are more abundantly and universally expressed than FLNC, also bound PC2. Indeed, the C-terminus of human FLNA (FLNAC, aa 2150-2647), FLNB (FLNBC, aa 2105-2602) and FLNC (FLNCC, aa 2144-2725) associated with both PC2N and PC2C as well (Fig. 1C).

**Figure 3:**
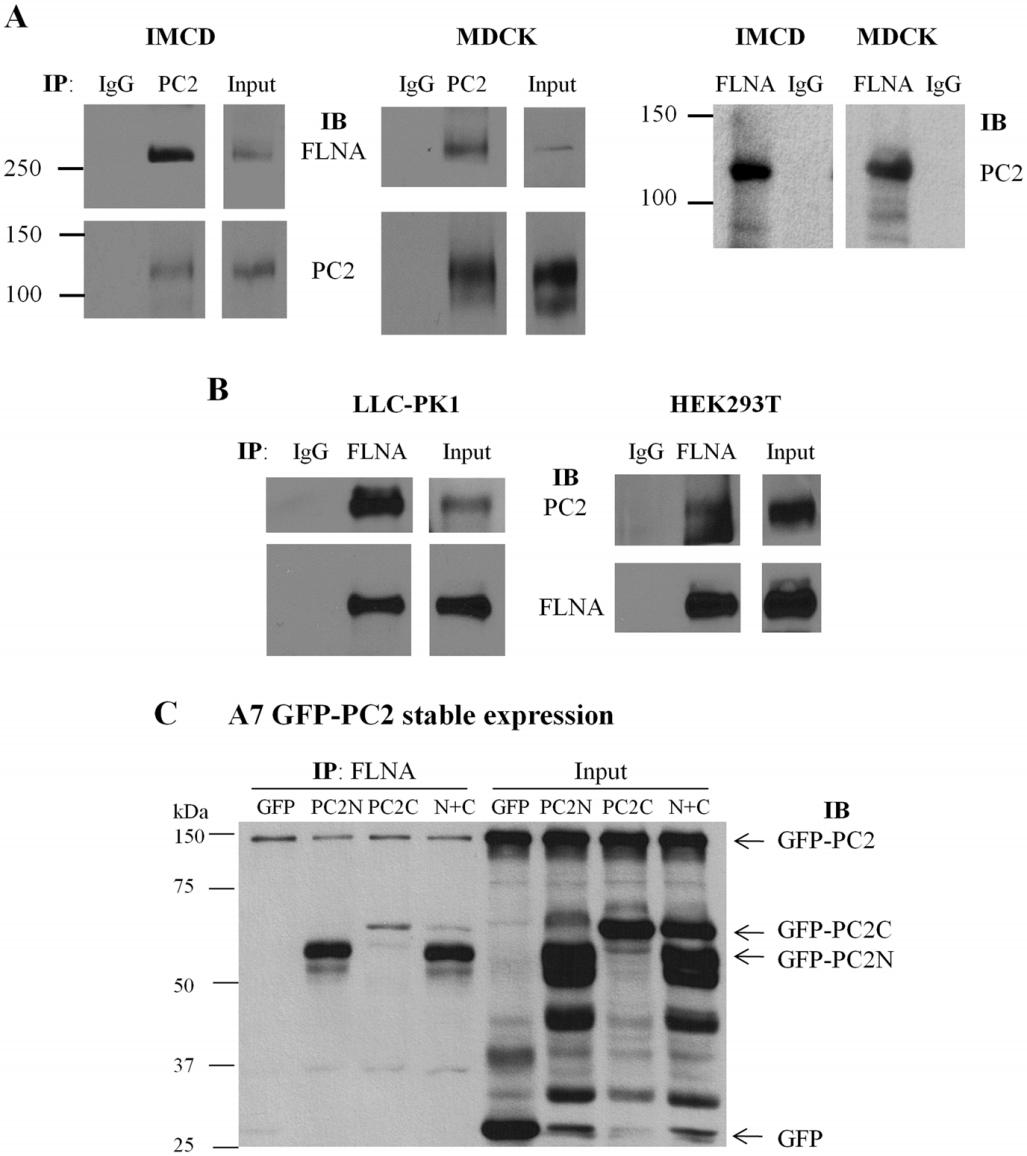
Interaction between PC2 and filamin by co-IP. (**A**) Interaction between endogenous PC2 and FLNA in IMCD and MDCK cells was determined by co-IP. Total proteins were precipitated with either anti-PC2 H-280 or non-immune rabbit IgG, and detected with the anti-FLNA antibody H-300 or anti-PC2 antibody 1A11 (Bound). Input indicates the protein expression of FLNA and PC2 (Total). Reciprocally, total proteins were precipitated with either anti-FLNA H-300 or non-immune rabbit IgG, and probed with anti-PC2 antibody 1A11. (**B**) Interaction between endogenous PC2 and FLNA in LLC-PK1 and HEK293T cells by co-IP. Total proteins were precipitated with either H-300 or non-immune rabbit IgG, and probed with anti-PC2 antibody 1A11 or H-300 (Bound). Input indicates the protein expression of PC2 and FLNA (Total). (**C**) Interaction of endogenous FLNA with over-expressed PC2 in A7 cells stably expressing GFP-PC2 and transiently expressing GFP-PC2N, GFP-PC2C, GFP-PC2N+GFP-PC2C (N+C), or GFP. After 48 hr of transient transfection, cells were collected for IP with anti-FLNA antibody (H-300) and the subsequent precipitates were subject to SDS-PAGE and immunoblotting with anti-GFP antibody (B-2) to detect the signals of GFP-PC2, GFP-PC2N, GFP-PC2C and GFP.

### Interaction of PC2 with Filamins Revealed by GST Pull-down and Co-IP

We employed an *in vitro* GST fusion protein affinity binding assay to further characterize the interaction between PC2 and FLNs. Both PC2N and PC2C were first fused in frame with a GST epitope, expressed in the bacterial strain BL21 in the presence of IPTG (1 mM), and then purified (Fig. 2A). His-tagged FLNAC, FLNBC and FLNCC were similarly expressed and purified (Fig. 2A). PC2N and PC2C present in the cell lysates were found to interact with purified FLNAC, FLNBC and FLNCC (Fig. 2B). Purified PC2N and PC2C also interacted with all three purified FLN C-termini (Fig. 2C). These data together demonstrated that PC2 directly binds the C-termini of filamins through their N- and C-termini. The amounts of PC2N pulled down by GST antibody was much lower than that of PC2C (Fig. 2B, GST bands, lower panels) whenever cell lysates were used. However, their binding to filamins was comparable (Fig. 2B, His bands, upper panels), indicating a stronger PC2N-filamin interaction than the PC2C-filamin interaction. Consistently, after taking into account the different amounts of purified PC2N and PC2C that were pulled down by GST antibody (Fig. 2C, GST bands, lower panels), it can be seen that the PC2N-filamin binding was stronger than the PC2C-filamin binding (Fig. 2C, His bands, upper panels). Thus, these data together indicated that binding of PC2N to the filamin C-terminus is stronger than that of PC2C to the filamin C-terminus.

**Figure 4:**
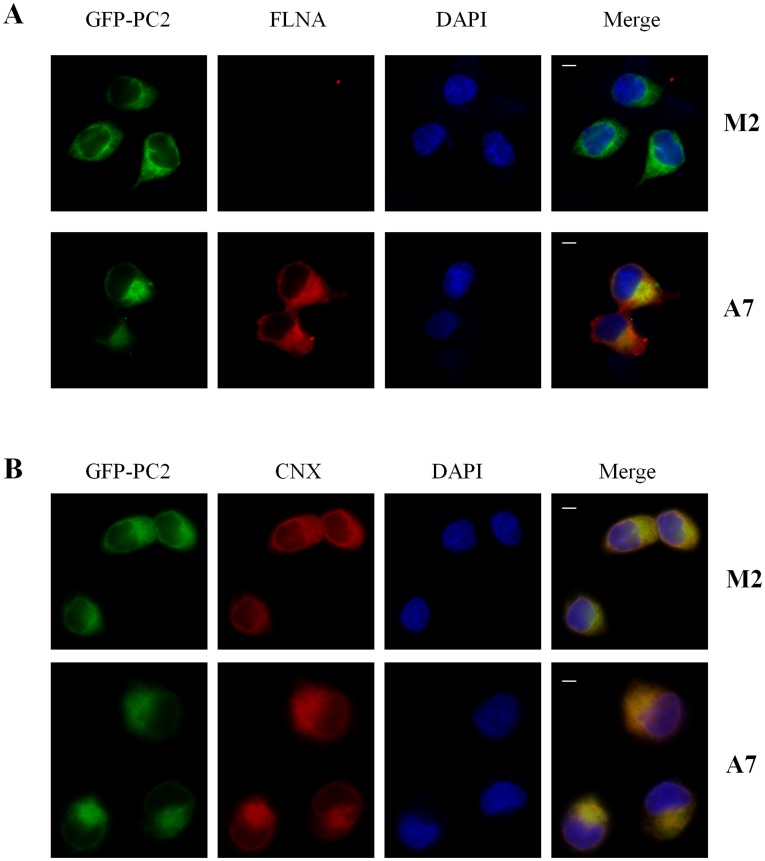
Cellular localization of PC2. M2 and A7 human melanoma cells over-expressing GFP-PC2 were grown on coverslips and incubated at 4°C overnight after fixation, with either anti-FLNA E-3 or anti-calnexin primary antibodies. The length of the white bar is 20 µm. (**A**) Subcellular co-localization of PC2 and FLNA. (**B**) Subcellular co-localization of PC2 and the ER marker calnexin (CNX).

To further explore whether endogenous PC2 interacts with FLN in mammalian cells, we performed co-IP experiments using IMCD and MDCK epithelial cells. FLN was detected in the precipitated lysates using a PC2 antibody, but not in the control immunoprecipitates using non-immune serum (Fig. 3A). Reciprocally, PC2 was detected in the FLN precipitated lysates (Fig. 3A). The endogenous PC2-FLN interaction was confirmed by co-IP in LLC-PK1 and HEK293T cells (Fig. 3B). Taken together, these results demonstrated that PC2 not only directly binds FLNs *in vitro* but also forms protein complexes with FLNs *in vivo*. Further, by over-expressing PC2N and PC2C in human melanoma A7 cells we found by co-IP that both PC2N and PC2C interact with endogenous FLNA and that the PC2N-FLNA interaction is stronger than the PC2C-FLNA interaction (Fig. 3C), in agreement with our results obtained from *in vitro* binding data (Fig. 1 and 2).

### Co-localization of PC2 with Filamin-A and Calnexin Revealed by IF

We stably expressed GFP-tagged human PC2 in FLNA-deficient M2 human melanoma cells, and A7 cells genetically rescued by expression of FLNA. The subcellular distribution of both PC2 and FLN was examined, and as expected, GFP signals were detected in both M2 and A7 cells, while FLNA was only observed in A7 cells (Fig. 4). We found that GFP-PC2 and FLNA partially colocalized in the perinuclear region of A7 cells (Fig. 4A). Using an antibody against the ER membrane marker calnexin, we also found that a significant fraction of intracellular PC2 localizes to the ER (Fig. 4B).

**Figure 5:**
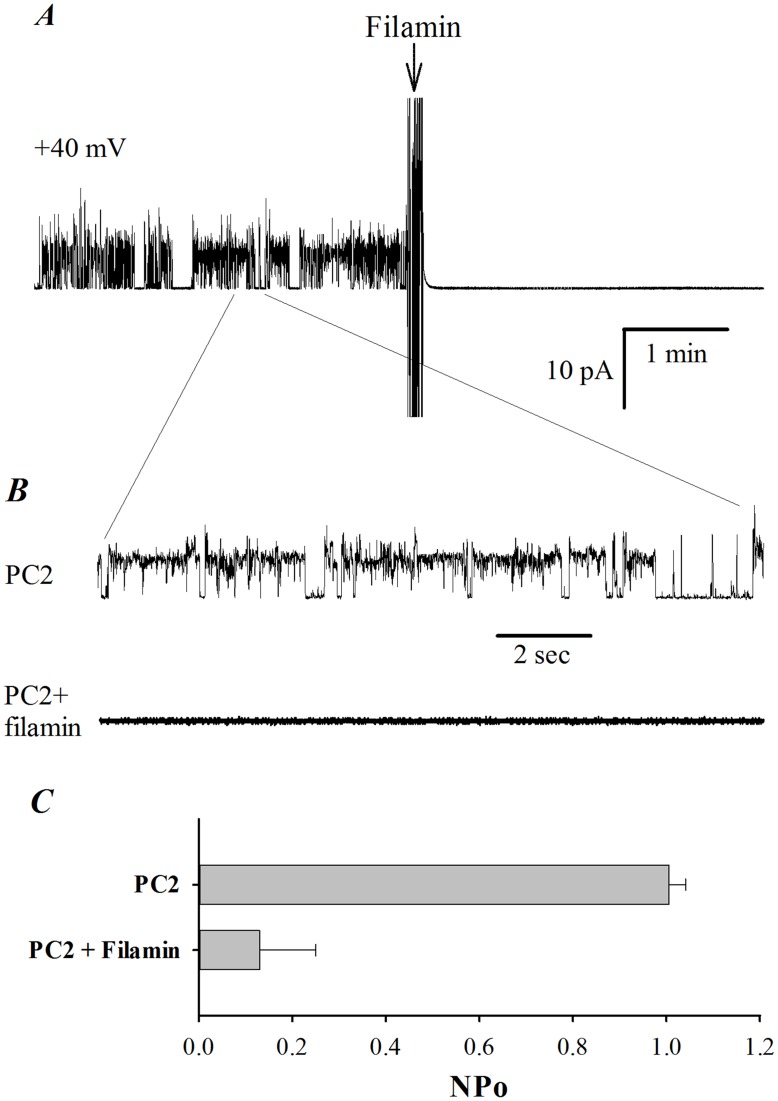
Effect of FLNA on purified PC2 channel activity in a lipid bilayer system. The PC2 protein was prepared by *in vitro* translation and reconstituted in a lipid bilayer system, in the presence of a KCl chemical gradient. (**A**) Representative tracings of reconstituted PC2 at +40 mV before and after addition of commercial chicken gizzard FLNA (25 nM), to the *cis* chamber. The *cis* (intracellular) compartment contained 10 mM MOPS, 150 mM KCl and 15 µM Ca^2+^ (by 1 mM EGTA and 1.01 mM/L CaCl_2_), pH 7.4 (adjusted by Tris-base). The *trans* (extracellular) chamber contained 10 mM MOPS and 15 mM KCl, pH 7.4. Tracings are representative of four experiments. (**B**) Expanded tracings from panel A, recorded before and after FLNA addition. (**C**) Averaged open probability recorded at +40 mV before and after addition of FLNA (N = 4, p<0.01, by paired t-test).

**Figure 6:**
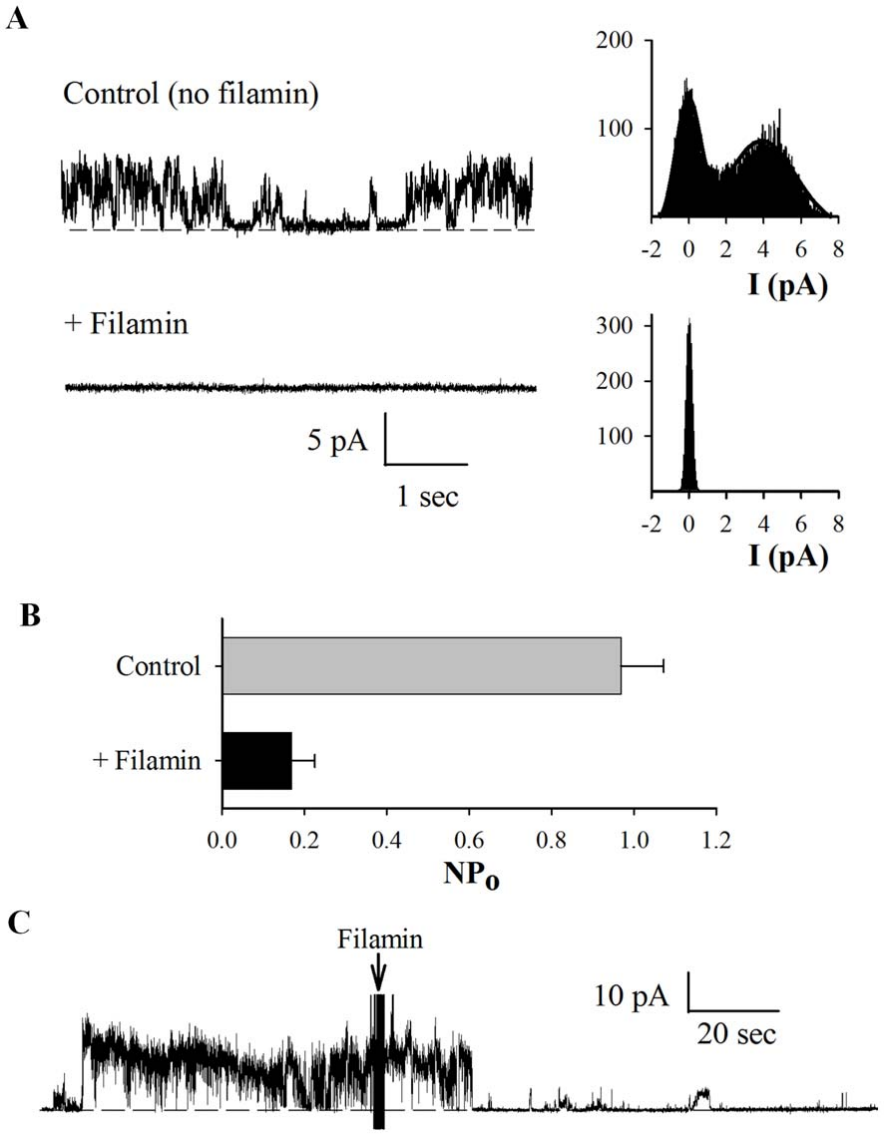
Regulation of hST PC2 channel function by FLNA. Apical hST membranes, containing endogenous PC2 were reconstituted in a lipid bilayer electrophysiology system, as in Fig. 5. (**A**) Representative recordings of PC2 channel activity in the absence and presence of commercial FLNA (25 nM) added to the *cis* chamber. All-point histograms are shown on the right of each tracing, to indicate current amplitude. Data are representative of seven experiments. (**B**) Averaged open probability in the presence and absence of FLNA (N = 7). Bars were statistically different, with p<0.05. (**C**) Representative recording showing real-time inhibition of PC2 activity by addition of FLNA to the *cis* chamber.

### Effect of Filamin on the Channel Function of PC2 Reconstituted in Lipid Bilayers

Among the various interactions between the actin cytoskeleton and PC2, previous studies have shown that both actin-binding proteins and actin cytoskeletal dynamics control PC2 channel function. Thus, we next explored the possibility that FLNA exerts a direct effect on the cation channel activity of PC2. For this, we used either PC2 protein obtained by *in vitro* translation, which should be devoid of associated proteins, or obtained from MDCK cells stably expressing PC2 by our modified tandem affinity purification method [27] (data not shown). PC2 was reconstituted in a lipid bilayer system for electrophysiology studies. We observed that addition of commercial chicken gizzard FLNA (25 nM) to the *cis* side of the lipid bilayer chamber abolished PC2 channel activity (N = 4, Fig. 5). In control experiments, addition of either the same saline solution containing no FLNA or denatured (boiled for 7 minutes) FLNA (25 nM) did not significantly affect PC2 channel activity (N = 4, data not shown), indicating the specific inhibitory effect of FLNA on PC2 channel. To provide further evidence of the inhibitory effect of FLN on PC2 channel function, we also used hST apical membrane vesicles expressing abundant endogenous PC2, as previously described [24], in a lipid bilayer reconstitution system. We observed that commercial chicken gizzard FLNA, but not the denatured one, also reduces PC2 channel activity in hST vesicles (Fig. 6).

## Discussion

In this study we have demonstrated, by various protein-protein interaction methods including yeast two-hybrid screen, GST pull-down and co-IP, that PC2 physically interacts with the actin cross-linking protein filamin. We also determined by a lipid bilayer electrophysiology reconstitution system that filamin functionally interacts with, and inhibits the channel function of PC2.

PC2 has been shown to interact with a number of components of both actin-based and microtubular cytoskeletons, including Hax-1 [30], CD2-associated protein [31], [32], tropomyosin-1 [29], troponin-I [33], α-actinin [21] and the kinesin-2 motor subunits KIF3A and KIF3B [25], [34]. Some of these interactions are not only structural, ie, helping anchorage of the channel protein to the cytoskeletal network, but also functional, enabling the regulation of PC2 channel activity. KIF3B, for example, was shown to mediate not only the physical interaction between PC2 and fibrocystin, the single transmembrane receptor-like protein mutated in human autosomal recessive PKD, but also the stimulation of the PC2 channel activity by fibrocystin [25]. On the other hand, KIF3A only binds PC2 but not fibrocystin, and directly stimulates PC2 channel activity [34]. An important interaction between the actin cytoskeleton and PC2 is observed by actin-binding proteins such as α-actinin. α-actinin not only binds PC2 but up-regulates its channel function as well. This may imply a more general regulatory mechanism in the control of ionic membrane permeability, because α-actinin also regulates the activity of a number of other ion channels, including the K^+^ channel Kv1.5 [35], the L-type Ca^2+^ channel [36] and NMDA receptors through direct binding [37], [38]. Here we have shown that FLN, an actin cross-linking protein that helps creating and regulating actin three-dimensional gels, binds directly to PC2N and PC2C, and represses PC2 channel activity. In this study, we employed several approaches to demonstrate structural and functional interactions between PC2 and the three isoforms of FLN. We also investigated the protein fragments of both PC2 and FLN that are involved in this interaction.

Interestingly, it was recently observed that PC2 expression inhibits stretch activated channels (SACs) activity in smooth muscle cells, while polycystin-1, a membrane receptor-like protein mutated in about 80% of ADPKD, reverses the inhibition by forming a protein complex with PC2 [39]. This study, which is believed to be important for understanding myogenic regulation, also seems to have revealed a potential physical interaction between PC2 and FLNA. Sharif-Naeini et al. [39] demonstrated that in the mouse VSMC line MOVAS, the actin cytoskeleton is indeed implicated in SAC inhibition by PC2, as this effect was abolished by F-actin disruption in the absence of FLNA. SAC nonselective channel activity was reduced in the presence of FLNA, and the inhibitory effect of PC2 expression was abolished when FLNA was absent. The study employed co-IP to show that PC2 and FLNA are in the same complex, since FLNA precipitated PC2 from COS cells over-expressing both PC2 and FLNA. It remained undetermined, however, not only as to whether the two proteins would bind each other directly, but also as to which domains were involved in the interaction and whether endogenous PC2 and FLNA would interact with each other *in vivo*. Our present study specifically answered these questions by using various *in vitro* and *in vivo* protein-protein interaction approaches (Fig. 1–3).

FLNs directly bind, mostly via their C-termini, to more than 20 protein partners with diverse functions, showing their great versatility as signalling scaffolds [2]. In particular, studies have demonstrated that FLN binding is important for the synthesis, surface membrane retention, and/or degradation of partner proteins. For example, FLNA directly binds furin to reduce its internalization and increase its protein synthesis [40]. Through physical binding, FLNA decreases the proteasomal/lysosomal degradation of the platelet glycoprotein subunit GpIbα, the G protein-coupled calcitonin receptor, and the class I IgG receptor FcγRI. FLNA also increases the degradation of epidermal growth factor receptor, and possibly of prostate-specific membrane antigen PSMA [41]–[45]. Finally, FLNA also stabilizes FcγRI and PSMA in the PM [40], [43], [45].

Although ADPKD is mainly associated with cyst formation in kidneys and liver, in the vascular system ADPKD and mutations in FLNs are associated with comparable manifestations, such as dissection, aneurysm and fragility [5]–[7], [14], [15]. It is thus important to determine the contribution of the PC2-filamin interaction to these vascular abnormalities, for example, through the use of animals with mutations in both PC2 and FLNs. In summary, our data indicate that the actin-cross-linking protein FLN is an interacting partner of PC2, and an important regulator of PC2 function, which may be relevant to the pathogenesis associated with mutations in either PC2 or FLNs.
